# ROS-sensitive calcipotriol nano-micelles prepared by methoxypolyethylene glycol (mPEG) – modified polymer for the treatment of psoriasis

**DOI:** 10.1080/10717544.2022.2086944

**Published:** 2022-06-24

**Authors:** Yulin Hua, Tiantian Chang, Kun Jiang, Jinhong Wang, Xiaodong Cui, Min Cheng, Fang Yan, Bo Song, Yuzhen Wang

**Affiliations:** a School of Pharmacy, Weifang Medical University, Weifang, China; b Basic Medical School, Weifang Medical University, Weifang, China

**Keywords:** Psoriasis, nano-micelle, calcipotriol, reactive oxygen species sensitive, transdermal drug delivery

## Abstract

Oxidative stress due to excessive reactive oxygen species (ROS) production in the skin microenvironment is one of the main mechanisms in psoriasis pathogenesis. A nano drug delivery system based on ROS-responsive release can enhance drug release at the target site. In this study, a ROS-sensitive material methoxypolyethylene glycol-thioether-thiol (mPEG-SS) was synthesized using mPEG as the parent structure with sulfide structural modification. An mPEG-SS-calcipotriol (mPEG-SS-CPT, PSC) nano-micelle percutaneous delivery system was prepared by encapsulating CPT. A small animal imaging system was used to study PSC’s the ROS-sensitive drug release process. It is shown that endogenous ROS mainly affects PSC and releases drugs. Finally, the therapeutic effect of PSC on psoriasis was explored by animal experiments. Ultimately, it ameliorates imiquimod-induced psoriasis-like inflammation. Overall, PSC is an effective ROS-sensitive transdermal drug delivery system that is expected to provide a new strategy for treating psoriasis.

## 1. Introduction

Psoriasis is one of dermatology's major research and disease control priorities worldwide (Armstrong & Read, 2020). Psoriasis develops regardless of age, is recalcitrant, is prone to relapse, and increases the risk of cardiovascular disease, metabolic syndrome, and psoriatic arthritis, severely affecting patients' physical and mental health (Luo et al., 2020; Parisi et al., 2020).

Oxidative stress due to oxidative and antioxidant systems imbalance in the human body is one of the primary mechanisms in psoriasis pathogenesis (Lin & Huang, 2016). The skin, a potential target of oxidative damage, is exposed to oxidative damage before forming the inflammatory microenvironment in psoriasis (Ali et al., 2016). Many studies have shown significantly elevated oxidative stress markers in psoriasis patients (Lin & Huang, 2016). Keratinocytes, fibroblasts, T cells, and neutrophils in the skin lesions of psoriasis patients produce excessive amounts of reactive oxygen species (ROS), including singlet oxygen (O_2_), hydroxyl radicals (OH^-^), superoxide anions (O^2-^), and hydrogen peroxide (H_2_O_2_) (Zorov et al., 2014; Zhu et al., 2021). ROS are produced in the mitochondria and released into the cytoplasm, inducing inflammatory factors release, DNA modification, and oxidative damage (Cannavò et al., 2019; Yu et al., 2020). Excessive ROS accumulation decreases the physiological antioxidant capacity of the skin and impairs the redox system of the epidermal microenvironment (Bito & Nishigori, 2012). Moreover, ROS are considered pro-inflammatory factors and signaling molecules to multiple metabolic pathways, overactivation of inflammatory signaling mechanisms in skin cells, and inducers of inflammatory cytokines (e.g. IL-6, IL-17, IL-22, and IL-8), keratinocytes, vascular endothelial cells, and chemokines which exacerbate the inflammatory response and create an inflammatory storm (Dainichi et al., 2018). In short, the use of antioxidants to regulate redox homeostasis and suppress psoriasis inflammatory responses is considered an effective therapy (Niki, 2014; Keum et al., 2020). However, studies on multifunctional nano-transdermal drug delivery systems with ROS-sensitive drug release and oxidative stress modulation for psoriasis treatment are still unreported.

In recent years, ROS-sensitive materials have become a hot research topic as delivery agents with controlled delivery and smart release functions. For example, sulfide groups were the first ROS-responsive groups applied in delivery systems, where hydrophobic sulfide fragments are oxidized and transformed into hydrophilic sulfoxide or sulfone, causing a shift in the structure, spatial site resistance, and long-chain polymers conformation, leading to the encapsulated drug release (Napoli et al., 2004; Brubaker et al., 2015). Polyethylene glycol (PEG) is a widely used water-soluble polymer with good biocompatibility (Grad et al., 2020; Martin et al., 2020). Sulfide modification of PEG allows the preparation of drug delivery systems, such as micelles, vesicles, nanoparticles, and dendritic macromolecules, and the encapsulation of poorly water-soluble drugs (Tapeinos & Pandit, 2016; Retout et al., 2019). Meanwhile, the modified polymers possess amphiphilic properties that allow self-assembly to form polymeric micelles and improve the drug’s bioavailability by improving biocompatibility, reducing side effects, prolonging cycle time, and increasing solubility or drug loading (D'Souza & Shegokar, 2016; Sun et al., 2021).

Psoriasis is still predominantly treated with a long course of topical drugs (Pradhan et al., 2013). One vitamin D_3_ analog, calcipotriol (CPT), inhibits cellular DNA and keratin synthesis by binding specifically to the vitamin D receptor (VDR), allowing correction of abnormal keratinocytes proliferation and differentiation (Frieder et al., 2017) is used as a primary topical agent for psoriasis. Common topical dosage forms such as creams (0.005%) and liniment (0.005%) are currently available. Recently, it has been suggested that nanoscale transdermal drug delivery systems can more effectively penetrate the thickened stratum corneum to achieve better results in psoriasis treatment (Mahmoud et al., 2017; Song et al., 2020).

This study synthesized a ROS-sensitive material (mPEG-SS) by modifying the methoxypolyethylene glycol (mPEG) parent structure and adding hydrophobic thioether and thiol structures. Using the amphiphilic nature of mPEG-SS, drug-loaded CPT-encapsulated nano-micelles were prepared to explore ROS stimuli-responsive triggered drug release against the psoriasis skin microenvironment and provide a new strategy for treating psoriasis.

## 2. Material and methods

### 2.1. Materials

mPEG_1000_, 4-toluenesulfonyl chloride (TsCl), potassium thioacetate (KSAc), hydroxylamine, and tetrahydrofuran (THF) were purchased from J&K Scientific Ltd. Carbotriol (CPT) was purchased from Maclean's Reagent, and Imiquimod cream (IMQ) was purchased from Mingxin Pharmaceutical Co. The positive control drug was the commercially available calcipotriol scalp solution (Daivonex, LEO Pharma A/S). The Enhanced BCA Protein Assay Kit was purchased from Beyotime Ltd. Mice were purchased from Jinan Panyue Experimental Animal Breeding Co.

### 2.2. Synthesis

The mPEG-SS synthesis was similar to the method reported in the literature (Gupta et al., 2014). In brief, mPEG1000 (1.0 g, 1.0 mmol) was dissolved in 26.0 ml anhydrous CH_2_Cl_2,_ and toluene sulfonyl chloride (9.0 g, 50.0 mmol) was added slowly dropwise, and the reaction was stirred at 65 °C for 3 h and then continued overnight (24 h) at room temperature. The CH_2_Cl_2_ was removed by rotary evaporation, dissolved in 6.0 mL anhydrous pyridine, added dropwise to 15 mL of pyridine/methanol (2:1) solvent mixture containing 1.2 g (10.5 mmol) of potassium thioacetate under nitrogen protection, and 500 μL of triethylamine was added to continue the reaction for 12 h. The mPEG thioacetate was obtained by spin evaporation. The mPEG thioacetate (0.5 g) was dissolved in 17.0 mL of anhydrous methanol, and 1.6 g of hydroxylamine was added to form a saturated solution. Then 3.65 mL of 25% methanolic sodium methanol solution was added and stirred in a water bath at 60 °C for 1 h and then stirred at room temperature overnight. After neutralization with 9 mL of 5 N acetic acid and freeze-drying, all samples were dissolved in 4.2 mL of 0.1 M potassium phosphate buffer (pH = 8.4), 155 mg of dithiothreitol (DTT) was added, and the reaction was carried out at 65 °C for 40 min. The resulting toluenesulfonyl-mPEG1000 was purified by a dextran gel G-25 column (50 cm × 3 cm) using 0.1 N acetic acids as the eluent. Toluenesulfonyl-mPEG1000 (1.0 mmol, 0.5 g) was dissolved in 25 ml THF, and propylene sulfide (60.0 mmol, 4.68 mL) was added dropwise under nitrogen protection. After the reaction at 0 °C for 2 h, 2-iodoethanol (2.0 mmol, 0.4 g) was added to quench the reaction. The reaction was stirred overnight at room temperature, washed three times with methanol, and spun to obtain a colorless viscous, transparent liquid. After adding water, it was dissolved, lyophilized, and dialyzed in a dialysis bag (1000 Da) to obtain mPEG-SS.

### 2.3. Structural identification and characterization

The synthetic mPEG raw material, the mPEG-SH intermediate, and the mPEG-SS end product were identified by hydrogen spectroscopy using CDCl_3_ as a solvent in a nuclear magnetic resonance spectrometer (BRUKER AVANCE 400 MHz) (Zhang et al., 2017). The molecular weight was determined by mass spectrum (MS). The mPEG-SS solutions with or without H_2_O_2_ (0.01, 0.02, 0.03, 0.04, 0.05, 0.06, 0.07, 0.08, 0.09, 0.10, 0.20, 0.30, and 0.40 mmol/L) were prepared and added to the test cups. The test cups were kept at a constant temperature of 25 °C in the reaction bath. The hanging ring was cooled after removing the dirt with an alcohol blowtorch, and the surface tension of each sample was tested three times per group. The data were plotted, and the inflection point was the critical micellar concentration (CMC).

### 2.4. Preparation of drug-laded nano-micelle PSC

Due to the poor water solubility of CPT, 0.5 mg CPT and 10.0 mg mPEG-SS were dissolved in 1.0 ml THF before the preparation of the drug-loaded micellar PSC. The mixture was diluted into 10 ml of PBS by sonication. The sonication conditions were: sonication time 5 min, sonication amplitude 35%, probe diameter 3 mm, interval time 5 s − 5 s. The beaker containing the sample was maintained in ice water throughout the sonication process, and the temperature difference of the system did not exceed 20 °C. Finally, THF was removed by spin evaporation, and the remaining liquid was centrifuged and sonicated to obtain the PSC solution (El Mohtadi et al., 2020).

### 2.5. PSC characterizations

Zeta potential, particle size, and distribution of PSC were examined with a laser scattering instrument (Zetasizer Nano ZS90, Malvern). The microstructure was determined using the method reported by Song (2020). The copper mesh containing PSC was negatively stained with 2.0% phosphotungstic acid for 30 min, dried, and then observed and photographed by transmission electron microscopy (TEM, HITACHI, HT7700, Japan). The encapsulation efficiency (EE%) and drug loading rate (DL%) of PSC-encapsulated CPT were detected by HPLC (100 μL). Briefly, PSC (1.0 mL, dry weight 10.405 mg) was dissolved with an appropriate amount of CH_3_OH, vortexed, shaken, and centrifuged at 11,000–12,000 rpm for 10 min. A small amount of the precipitate was filtered (0.45 μm), and 10.0 μL of the filtrate was diluted with ethyl acetate (20.0 μL) and methanol (1.0 mL). The CPT concentration was calculated with Formulas 1 and 2 (Sonawane et al., 2014):

(1) $$\text{EE}(\%)=\frac{\text{Weight}\text{of}\text{loaded}\text{CPT}}{\text{Weight}\text{of}\text{initial}\text{CPT}}\times 100\%$$

(2) $$\text{DL}(\%)=\frac{\text{Weight}\text{of}\text{loaded}\text{CPT}}{\text{Total}\text{weight}\text{of}\text{PSC}}\times 100\%$$

### 2.6. *In vitro* oxidation-sensitive drug release

The as-prepared PSC nano-micelles were dispersed in PBS solution containing 50 μM H_2_O_2_ to assess their ROS sensitivity. The changes in PSC particle size at different times (0, 2, 4, 6, 8, 10, 12, 14, and 16 h) were examined by dynamic light scattering. To examine the drug release effect of PSC, mPEG-SS-encapsulated fluorescein diacetate (FDA) was prepared as mPEG-SS-FDA micelles (PSF) and incubated in 96-well plates (PBS solution containing 50 μM H_2_O_2_). Fluorescence intensity changes were observed under 490 nm and 526 nm excitation light using a small animal imaging system (PerkinElmer, IVIS Spectrum, USA) at the time points set above (An et al., 2020).

### 2.7. Cell viability assay

HaCaT cells (1 × 10^4^ cells/well) were seeded in 96-well plates for 24 h. The experimental group was incubated with DMEM containing PSC for 24 h. EdU solution (100 μL) was added to each well at a ratio of 1:1000. After 2 h of incubation, the cells were fixed in 4% paraformaldehyde (50 μL) for 30 min. Fifty microliters of 2.0 g/L glycine were added to each well and incubated in a decolorizer shaker for 5 min. The glycine solution was discarded, and 100 μL of 0.5% Triton-X was added to each well and incubated for 10 min. Apollo staining reaction solution (100 μL of 1 ×) was added to each well and incubated for 30 min at room temperature, avoiding light. The staining solution was discarded, and 100 μL of methanol and 100 μL of 4′, 6-diamidino-2-phenylindole (DAPI) staining solution were added for staining in the dark for 30 min. The cells were observed under a fluorescence microscope, and the cell proliferation rate was calculated based on the amount of EdU color development and cell nuclear fluorescence.

### 2.8. Extracellular ROS-scavenging activity

The PSC effect on extracellular ROS clearance was quantified using the ECL Western Blotting Detection kit. HaCaT cells were first seeded in 96 wells at a density of 2 × 10^4^ cells/well, and H_2_O_2_ was added to each well at a concentration of 50 μM. Then PSC was added and incubated for 4 h. The supernatant was removed by centrifugation. Then 50 μL of supernatant from each sample was added to the 96-well plate separately, and 50 μL PBS was added to bring the volume of each well to 100 μL. A mixture of reagents A and B from the ECL kit (40:1, v/v) was added to each well and incubated for 5 min at room temperature in the dark. The chemiluminescence values of the samples were measured at 430 nm under an enzyme marker (Thermo, USA) (Keum et al., 2020).

### 2.9. Intracellular ROS-scavenging activity

To examine the PSC scavenging of intracellular ROS, an mPEG-SS-encapsulated ROS fluorescent probe 2′, 7′-dichlorodihydro-fluorescein diacetate (DCFH-DA) was firstly prepared as mPEG-SS-DCFH-DA nano-micelles (PSD). DCFH-DA can be cleaved to 2′, 7′-dichlorofluorescin (DCFH) by cellular esterases after cellular uptake and oxidized to 2′, 7′-dichlorofluorescein (DCF) by ROS (Yao et al., 2015). HaCaT cells (4 × 10^4^ cells/well) were seeded in 12-well plates and incubated at 37 °C and 5.0% CO_2_ for 24 h. After 24 h, the medium was removed, the cells were incubated with the PSD-containing medium, and a serum-free medium containing 50 µM H_2_O_2_ was added after 24 h. After 6 h, DCFH-DA (10 µM) was added to the cells and incubated for another 20 min. The light intensity was detected at excitation light at 485 nm and 535 nm using an enzyme marker, and the cells were photographed under a fluorescence microscope (Gangadevi et al., 2021).

### 2.10. Animal experiments

#### 2.10.1. Modeling and grouping

All animal treatments and laboratory procedures were conducted according to the National Institutes of Health Guide for the Care and Use of Laboratory Animals (NIH Publications No. 8023, revised 1978) and approved by the Ethics Committee of Weifang Medical University (2018-103). In brief, psoriasis-like skin lesions were induced in mice with imiquimod (IMQ). The mice were depilated on the back (2.0 cm × 3.0 cm) and evenly coated with 5.0% IMQ cream (200 μL) once in the morning and once in the evening for 8 d. Thirty-six BALB/c mice (6-8 weeks old) weighing 20.0 ± 3.0 g were divided into healthy control, untreated model, mPEG-SS (0.1% w/v), free CPT (0.005% w/w), PSC (0.004% w/w), and positive control groups (Doivonex, 0.005% w/w). The treatment was started on day 9, and applied 200 μL drug solution twice daily for 8 days (Song et al., 2020).

#### 2.10.2. *In vivo* fluorescence

After successfully establishing the psoriasis mice model, PSF was applied to the skin lesions. Changes in fluorescence intensity were observed at 0, 2, 4, 8, 12, and 16 h with a small animal live imaging system to examine the *in vivo* drug release of nano-micelles (Khmaladze et al., 2014).

#### 2.10.3. PASI scoring

The area and severity of psoriasis-like lesions (PASI) were used as an index to observe the degree of erythema, scaling, plaques, and hypertrophy of the skin in the experimental areas of each group and scored according to a five-point scale (0 = none, 1 = mild, 2 = moderate, 3 = severe, and 4 = very severe). The mean value was taken for each group (*n* = 6) (Song et al., 2020).

#### 2.10.4. Histomorphology

Twenty-four hours after the last dose, the mice were euthanized, and the lesioned skin area was fixed in 10% formaldehyde solution, paraffin-embedded, sectioned (thickness about 0.5 mm), stained with H&E and VEGF staining kit (Solarbio, Beijing, China), and photographed (Wang et al., 2019).

#### 2.10.5. Intracellular ROS measurements in isolated primary keratinocytes

The collected dorsal skin tissue samples were incubated with 0.25% trypsin for 2 h at 37 °C in a shaker to isolate primary keratinocytes. Then, the samples were dissociated with a pipette, and trypsin was inactivated with a low-calcium DMEM medium containing 10% fetal bovine serum. The total number of cells isolated was counted and normalized. The cells were washed with PBS and then seeded in 96-well plates with 20 μM DCFH-DA (diluted in DMSO) per well and incubated for 0.5 h at 37 °C. Finally, an enzyme marker detected light intensity at 485 nm and 535 nm (Wang et al., 2019).

#### 2.10.6. Cytokine experiments

Blood was collected from the orbit of each mouse and left for 30 min to clot, centrifuged at 2500 rpm for 5 min at 25 °C, and the serum was collected and stored in a −80 °C refrigerator. IL-6 and TNF-α levels were measured according to the ELISA kits instructions (Wang et al., 2019).

### 2.11. Data analysis

Data were expressed as mean ± SD and analyzed by *t*-tests using Origin 9.0 software.

## 3. Results and discussion

### 3.1. Chemical structure and characterizations of polymers

**Figure 1.**
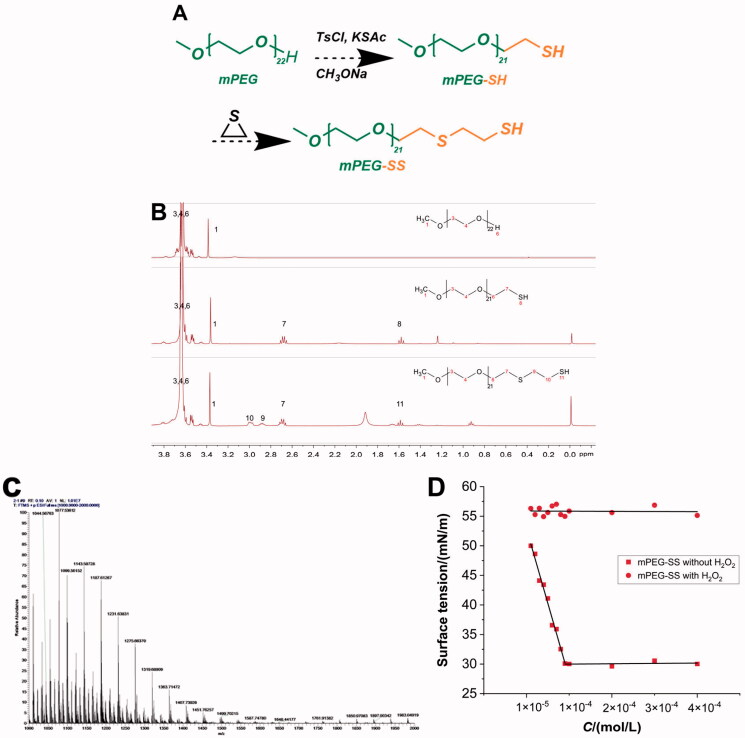
(A) Synthetic route of mPEG-SS; (B) ^1^H-NMR spectra of mPEG, mPEG-SH, and mPEG-SS; (C) MS for mPEG-SS; (D) CMC of mPEG-SS with and without H_2_O_2_.

The target compound mPEG-SS was synthesized and obtained by multi-step reaction (Figure 1A). ^1^H-NMR (400 MHz, CDCl_3_, δ): 3.36 (s, 3H, CH_3_-1), δ 3.52–3.81 (m, *J* = 7.1 Hz, 86H, OCH_2_-3,4,6,), δ 2.15-2.10 (m, 2H, SCH_2_-7), δ 2.81–2.89 (t, *J* = 16 Hz, 2H, SCH_2_-9), δ 2.91–3.00 (m, 2H, 10-CH_2_SH), δ 1.55–1.59 (t, *J* = 8 Hz, 1H, SH-11), with two more characteristic peaks of "9" and "10" than mPEG-SH (Figure 1B). The number of hydrogens, peak area and MS (Figure 1C) suggested that an ethylene sulfide group was added to the mPEG-SH structure. The CMC of mPEG-SS without H_2_O_2_ was 9.0 × 10^−5 ^mol/L (Figure 1D).

The synthesis of mPEG-SS is an anionic polymerization reaction initiated by thiol anions. mPEG is a hydrophilic polymer and an ethylene sulfide is a hydrophobic group, and their polymerization creates amphiphilicity, providing the structural basis for its micelle formation (Gupta et al., 2014; Wu et al., 2018). In addition, the lower CMC and the change after encountering H_2_O_2_ allow the formation and depolymerization of micelles. The strong hydrophobicity of the ethylene sulfide group improves the stability of the micelles (Napoli et al., 2004). The sulfhydryl group (-SH) has a weaker ability to form hydrogen bonds with water than the hydroxyl group (-OH) and, therefore, higher lipid solubility. CPT drugs are less water-soluble, self-assemble by affinity to the ethylene sulfide hydrophobic groups, and form micelles with a hydrophobic interior and a hydrophilic exterior. In addition, the ultrasound-assisted technique allows the drug-loaded micelles to reach the nanoscale. This greatly increases the specific surface area of the drug, which is beneficial for enhancing the ROS sensitivity of the material (Keum et al., 2020).

### 3.2. PSC characterizations and *in vitro* ROS-sensitive drug release

**Figure 2.**
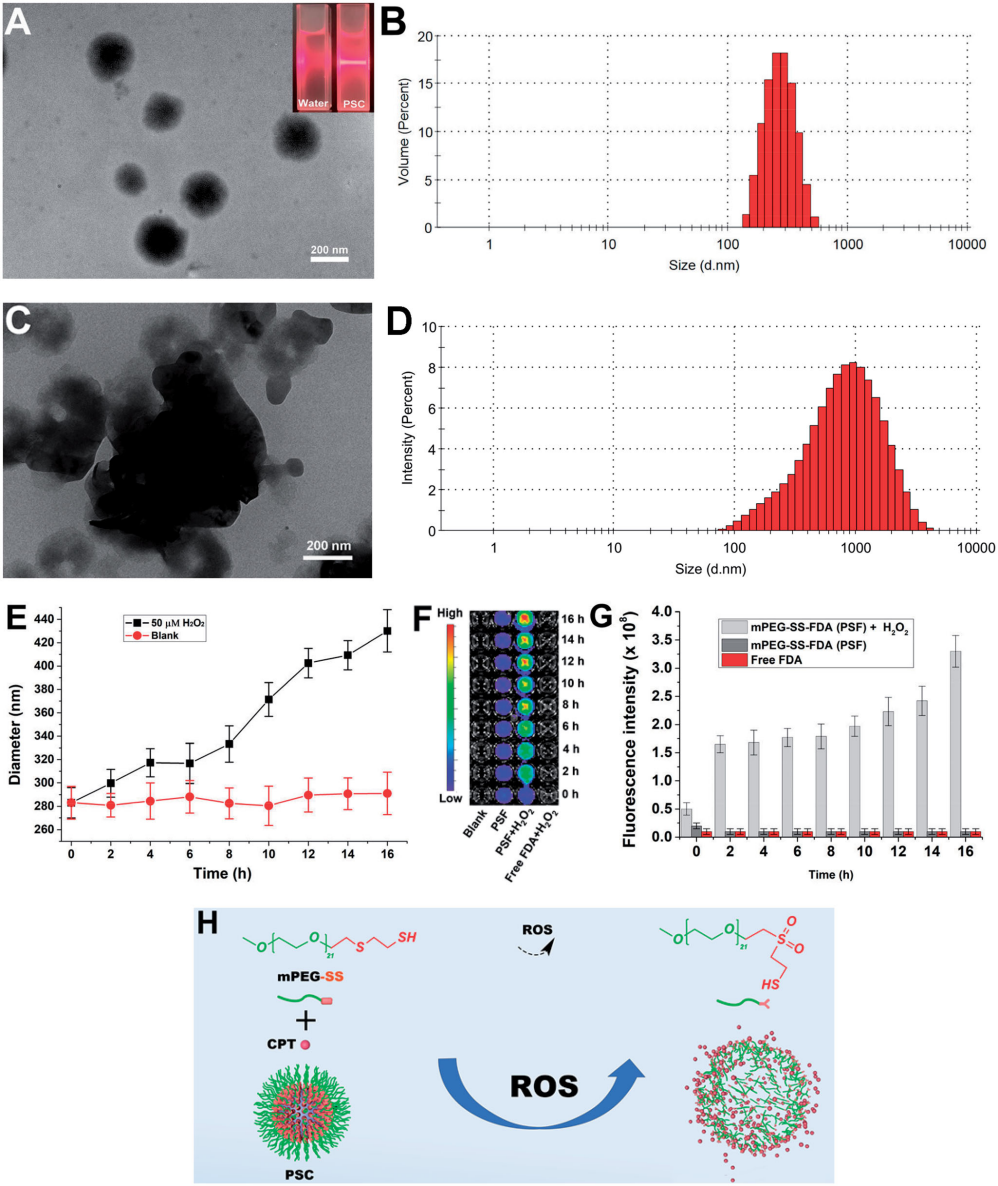
(A) Microstructure of PSC without H_2_O_2_ and Tyndall phenomenon (upper right corner) of PSC; (B) Particle size distribution of PSC without H_2_O_2_; (C) H_2_O_2_ effect on PSC microstructure (24 h); (D) H_2_O_2_ effect on PSC particle size; (E) Particle size curve with time; (F) Time-dependent fluorescence of PSF incubated without and with H_2_O_2_; (G) Histogram of time-dependent fluorescence; (H) Depolymerization schematic diagram of PSC.

The prepared PSC showed the Tyndall phenomenon (Figure 2A, upper right corner). The EE% and DL% of PSC were approximately 80.91% and 3.89%, respectively. Under H_2_O_2_ deprivation, the PSC was observed under TEM as rounded spheres (Figure 2A) with an average particle size of 283 ± 13.1 nm (Figure 2B) and a Zeta potential value of −14.9 mV. When PSC was dispersed in PBS containing 50 μM H_2_O_2_ for 24 h, the PSC underwent disassembly (Figure 2C), and the particle size became progressively larger (Figure 2D,E). The ROS-triggered release was studied by FDA-loaded nano-micelles (PSF) as a model drug and as an alternative to hydrophobic drugs. The imaging results (Figure 2F,G) show that the fluorescence intensity increases with time under H_2_O_2_ conditions, indicating that the PSF releases the fluorescent FDA continuously. In contrast, under the H_2_O_2_ deprivation, the FDA fluorescence was not evident due to fluorescence quenching. The above results indicate that PSC has some ROS sensitivity and can release drugs.

The ROS effect on the PSC particle size reflects micelles' disintegration process. The thioether bond (-S-) in the mPEG-SS structure cannot form hydrogen bonds with water, so the -S- containing end is more hydrophobic. The hydrophobic -S- is oxidized to the more hydrophilic sulfoxide or sulfone, and the conformation of the polymer, the spatial site resistance of the group, and the hydrophobicity are changed, allowing the micelles to depolymerize and release the carried drug (Figure 2H). Most of the known synthetic ROS-sensitive materials are sensitive to H_2_O_2_ at concentrations ranging from 1 μM to 10000 μM, and the sensitivity is proportional to time. In contrast, the H_2_O_2_ level *in vivo* in the pathophysiological state is about 50–100 μM (Tapeinos & Pandit, 2016). The sensitive concentration of PSC to H_2_O_2_ is in line with physiological ROS levels, providing a possibility for its *in vivo* application.

### 3.3. Cytotoxicity

**Figure 3.**
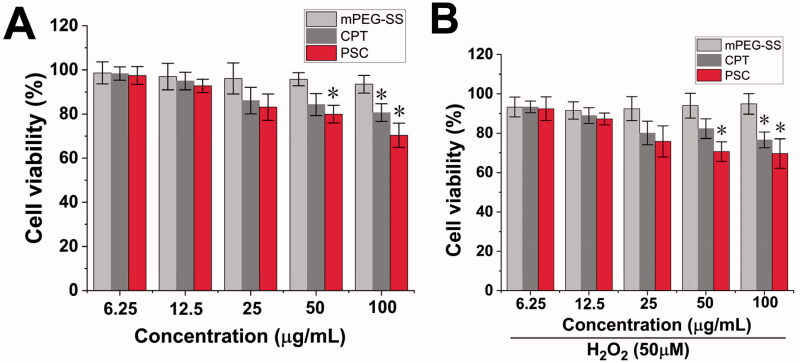
(A) Cytotoxicity of mPEG-SS, CPT, and PSC on HaCaT without H_2_O_2_; (B) Cytotoxicity of mPEG-SS, CPT, and PSC on HaCaT under H_2_O_2_ condition (*P < 0.05 compared with the control group, *n* = 3).

The mPEG-SS, CPT, and PSC effects on HaCaT cell viability were detected by the EdU method. Figure 3A,B showed that the cell viability of the mPEG-SS group did not change significantly within 24 h with or without H_2_O_2_ treatment, indicating that the materials were safe for HaCaT cells. In contrast, there was a significant decrease in cell viability in the PSC and CPT groups at concentrations greater than 50 μg/mL, which may come from the CPT toxicity, so it is necessary to control the CPT concentration.

PEG has been accepted in the pharmacopeias of several countries and is a nontoxic, nonirritating, and biocompatible material for pharmaceutical use (Keum et al., 2020). There have been numerous reports on the synthesis and modification of PEG as a parent structure. For example, An (2020) modified PEG with sulfide repeating units and then encapsulated Celastrol to treat arthritis (An et al., 2020). Modified -SH groups are commonly found in the human body, and endogenous substances, such as reduced glutathione (G-SH), contain -SH bonds. G-SH is a physiological antioxidant in the body and one of the main cellular barriers against oxidative stress (Gu et al., 2015; Diao et al., 2020). G-SH can be used as an antidote, antioxidant, and excipient in functional foods, such as immune-enhancing or antitumors (Lee et al., 2020). This provides information based on mPEG-SS safety.

### 3.4. ROS-scavenging activity

**Figure 4.**
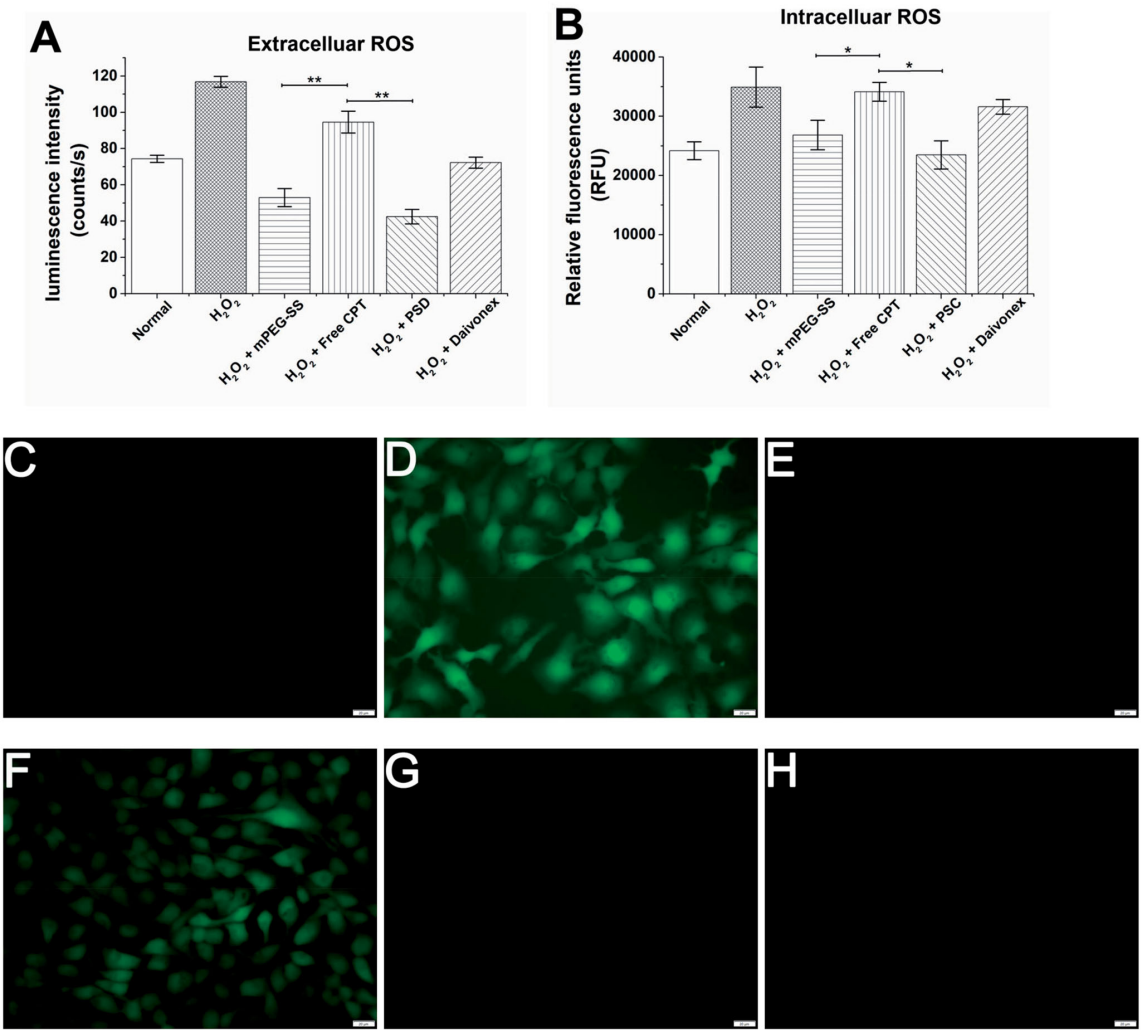
(A) PSC scavenging extracellular ROS; (B) PSC scavenging intracellular ROS; (C) Cellular images of PSC scavenging intracellular ROS of the normal group; (D) H_2_O_2_ group; (E) H_2_O_2_ + mPEG-ss group; (F) H_2_O_2_ + free CPT group; (G) H_2_O_2_ + PSD; (H) Positive control group; (scale bar: 20 μm; **P < 0.01; *P < 0.05 compared with the control group, *n* = 3).

Using HaCaT as an *in vitro* cell model, the scavenging effect of PSC on extracellular ROS was quantified by the ECL kit. Figure 4(A) showed that the mPEG-SS and PSC groups exhibited a better scavenging effect, while the free CPT group showed an insignificant effect. The PSC scavenging effect on intracellular ROS was examined using 2′, 7′-dichlorofluorescein as a fluorescent probe (Figure 4B–H). The fluorescence intensity in the PSC and mPEG-SS group cells was significantly reduced, indicating that both could scavenge intracellular ROS. These results suggest that PSC has a scavenging effect on extracellular and intracellular ROS, which is highly beneficial for redox balance regulation in the skin microenvironment.

The extracellular and intracellular ROS scavenging effects were mainly from mPEG-SS. The -S- in the mPEG-SS structure is readily oxidized and consumes ROS, resulting in lower ROS levels in the internal environment. ROS are mainly derived from mitochondria and are by-products of cellular respiration and protein folding or end products of many metabolic reactions (Zorov et al., 2014; Yu et al., 2020). ROS are released by mitochondria into the cytoplasm and continuously diffuse outside the cell, and excessive accumulation leads to mitochondrial dysfunction or damage and imbalance of the cellular antioxidant mechanisms (Nickel et al., 2014; Oyewole & Birch-Machin, 2015). Inflammatory cells in psoriasis lesions can produce excessive ROS and various inflammatory factors that cause abnormal keratinocyte proliferation and excessive thickening of the stratum corneum (Bito & Nishigori, 2012). Therefore, effective ROS scavenging may improve psoriasis by protecting skin cells from oxidative damage.

### 3.5. *In vivo* drug release and therapeutic effect in psoriasis animal model

**Figure 5.**
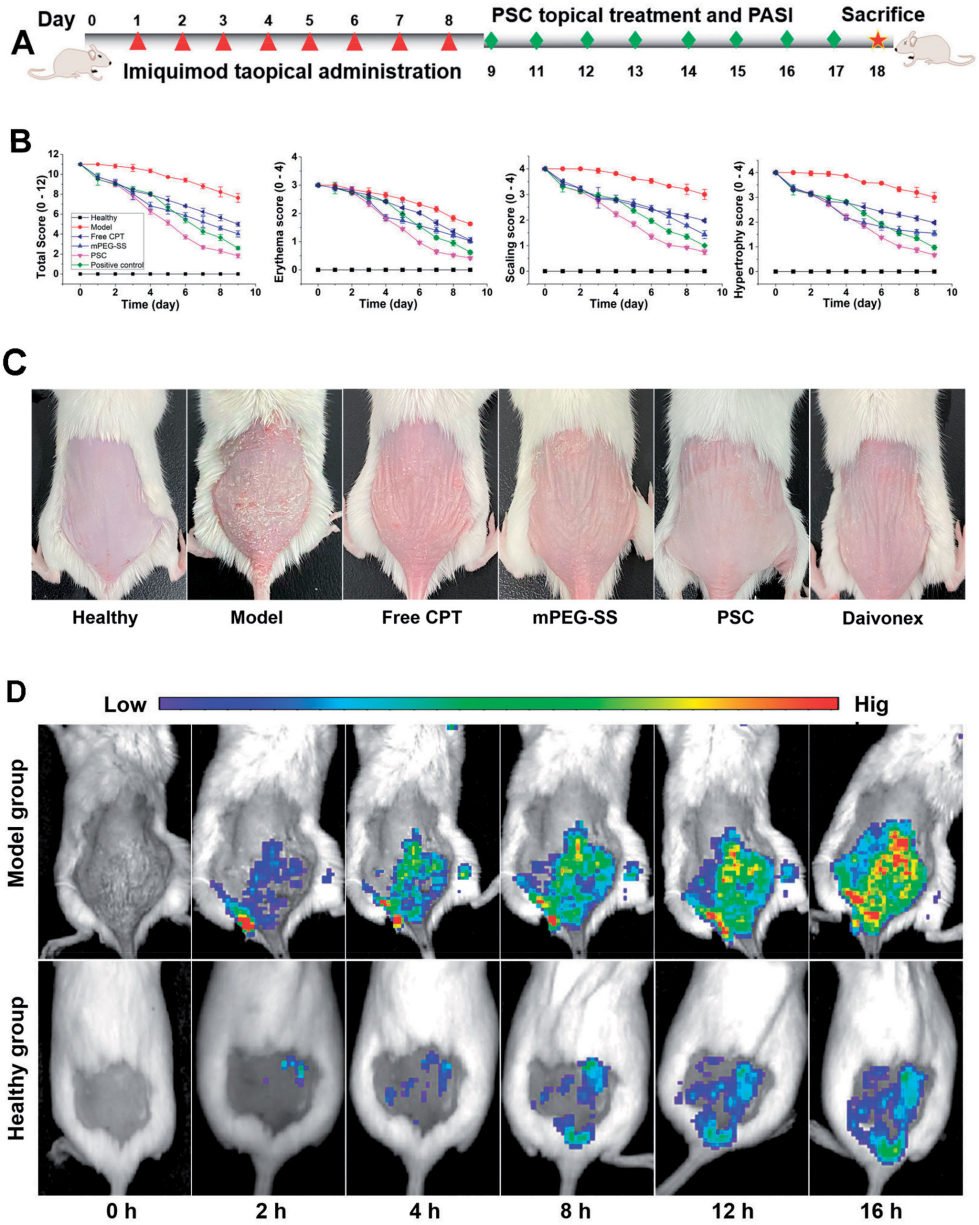
(A) Molding and drugging procedure of psoriasis mice; (B) PASI score (total, erythema, scaling, hypertrophy score); (C) Photographs of dorsal skin of each group of mice; (D) *In vivo* fluorescence imaging.

During the modeling process (9 d) (Figure 5A), the model group mice showed more severe scaling and erythema, incomplete epidermal keratinization, and thickened and pestle-like hyperplasia of the stratum corneum (Figure 5B/C). After 8 d of treatment, the PASI scores of the free CPT and mPEG-SS groups gradually decreased; however, a small amount of scaling and erythema (Figure 5B,C). In contrast, in the PSC experimental group, various pathological features were significantly reduced, there was a small amount of hair regeneration, and a significantly shorter treatment period. In addition, the small animal imaging results also showed (Figure 5D) that the PSD fluorescence intensity on the skin of mice in the model group showed a gradual increase, indicating that the micelles were gradually disintegrating and releasing the fluorescent substances. In contrast, the fluorescence intensity trend in the normal group of mice did not change, indicating that the PSD was less depolymerized and that the micelles had certain *in vitro* stability.

IMQ is a toll-like receptor (TLR 7/8) agonist that induces erythema, scaling, keratinocytes proliferation with echinodermatous, keratosis imperfecta, and T-cell infiltration in the skin of mice with phenotypic features similar to human psoriasis and is recognized as a modeling tool (Van der Fits et al., 2009; Chong et al., 2017). This study showed that PSC reduced pathological symptoms such as erythema and scaling. Animal imaging results show that endogenous ROS mainly affects PSC. ROS are generated from mitochondria and continuously diffuse outside the cell and into the internal environment, where they accumulate in excess and lead to oxidative stress (Zorov et al., 2014). Nanoscale PSCs are more likely to penetrate the skin and appendages and improve drug delivery efficiency and resistance to oxidative stress.

### 3.6. Histomorphology and inflammatory factors

**Figure 6.**
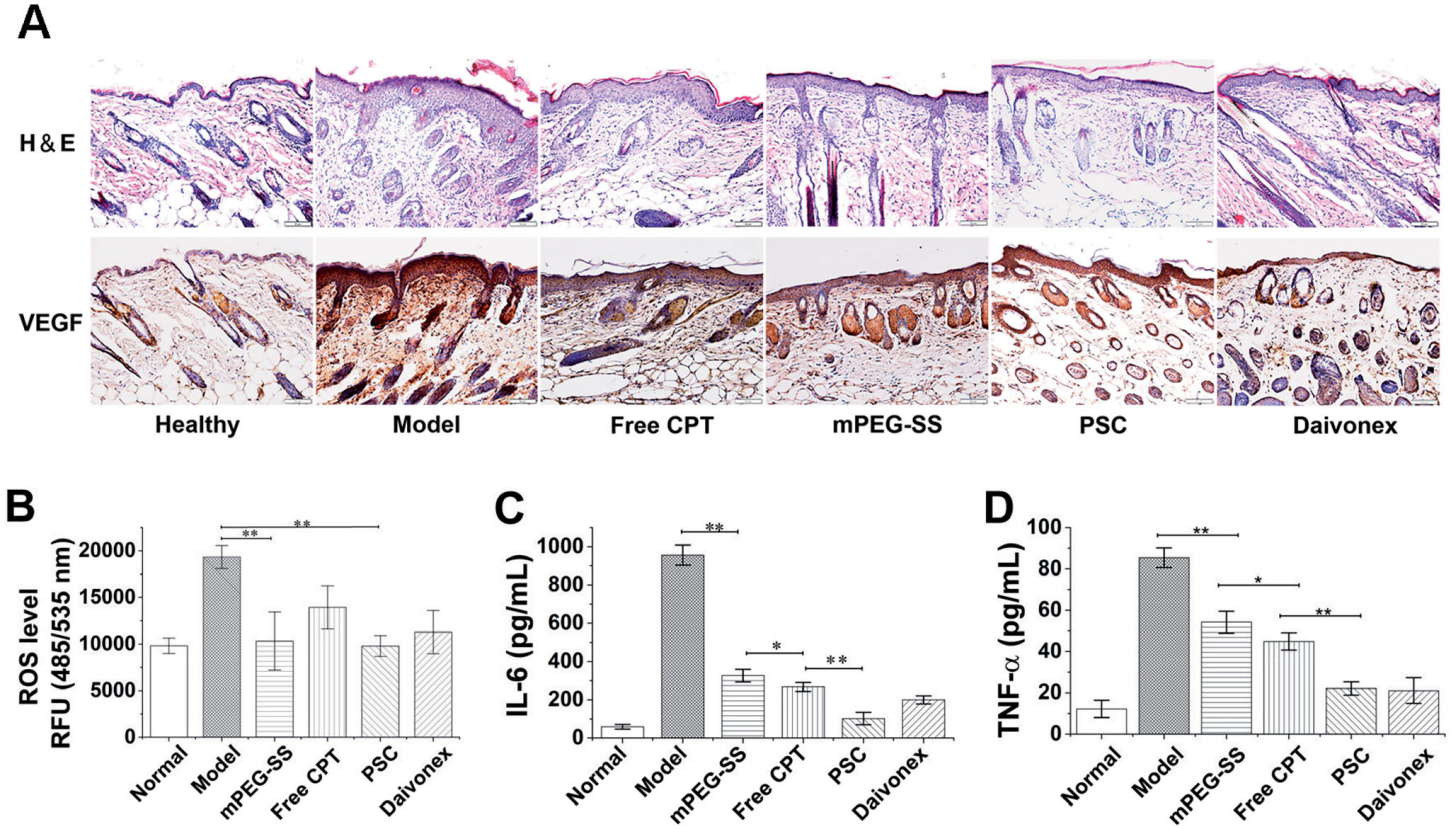
(A) H&E and VEGF staining; (B) *In vivo* ROS-scavenging effect; (C) Serum IL-6; (D) TNF-α level (**P < 0.01; *P < 0.05 compared with the control group, *n* = 3).

The H&E staining of the PSC group showed a significant reduction of inflammatory cell infiltration at the lesion compared to the positive control group (Figure 6A). Simultaneously, VEGF staining showed that VEGF expression was significantly elevated in the model group and significantly decreased in the PSC group (Figure 6A). The effect of PSC on ROS-scavenging *in vivo* was also noticeable (Figure 6B). The cytokine analysis showed that PSC could effectively reduce serum IL-6 (Figure 6C) and TNF-α levels (Figure 6D).

PSC reduced pathological symptoms, such as inflammatory cell infiltration and reduced microangiogenesis and VEGF expression. ROS induces VEGF expression in a variety of cells. Excessive ROS accumulation in the skin lesions of psoriasis mice drives the inflammatory factors release, including IL-6, VEGF, and TNF-α. Moreover, inflammatory cells are recruited and infiltrate the lesions, interacting with and promoting abnormal proliferation and differentiation of keratinocytes, exacerbating inflammation (Young et al., 2008). Studies in the interaction between oxidative stress and angiogenesis mainly focused on the VEGF signaling pathway (Hu et al., 2021). Therefore, the ROS-scavenging by PSC also affects VEGF reduction. In addition, *in vivo* ROS assay results indicate that PSC has a ROS-scavenging effect on skin lesions. The levels of inflammatory factors IL-6 and TNF-α were also significantly reduced.

## 4. Conclusion

Aiming at the oxidative stress mechanism caused by ROS overproduction in the psoriasis skin microenvironment, a ROS-sensitive material mPEG-SS was synthesized and a PSC nano-micelle percutaneous delivery system was prepared by encapsulating CPT. The advantages of ROS sensitivity, good biocompatibility, safe route of administration, and short treatment cycle demonstrated by PSC show great potential for the percutaneous psoriasis treatment. This study provides a new strategy for a ROS-sensitive nano-micellar drug delivery system in the percutaneous treatment of psoriasis. More research on the sensitivity of the ROS-responsive material shall be performed in the future.
